# When free air misleads: pneumoperitoneum from a ruptured pyogenic liver abscess

**DOI:** 10.1093/jscr/rjaf867

**Published:** 2025-11-04

**Authors:** Yavor Asenov, Boris Kunev, Todor Yanev, Branimir Golemanov, Plamen Getsov, Nikolay Penkov

**Affiliations:** Department of Surgery, University Hospital “Tsaritsa Yoanna–ISUL”, Medical University–Sofia, 1527 Sofia, Bulgaria; Department of Surgery, University Hospital “Tsaritsa Yoanna–ISUL”, Medical University–Sofia, 1527 Sofia, Bulgaria; Department of Surgery, University Hospital “Tsaritsa Yoanna–ISUL”, Medical University–Sofia, 1527 Sofia, Bulgaria; Department of Gastroenterology, University Hospital “Tsaritsa Yoanna–ISUL”, Medical University–Sofia, 1527 Sofia, Bulgaria; Department of Diagnostic Imaging, University Hospital “Tsaritsa Yoanna–ISUL”, Medical University–Sofia, 1527 Sofia, Bulgaria; Department of Surgery, University Hospital “Tsaritsa Yoanna–ISUL”, Medical University–Sofia, 1527 Sofia, Bulgaria

**Keywords:** pyogenic liver abscess, spontaneous rupture, pneumoperitoneum, *Enterococcus faecalis*, percutaneous drainage

## Abstract

Pyogenic liver abscess (PLA) is a severe condition with mortality rates of 10%–19%. Spontaneous rupture occurs in about 5% of cases and carries much higher mortality. Rarely, ruptured PLA presents with pneumoperitoneum, mimicking hollow viscus perforation. We report a 48-year-old man with chronic gastric ulcer disease who presented with fever, malaise, nausea, and abdominal distension two weeks after endoscopic hemostasis. He rapidly deteriorated with septic shock and peritoneal signs. Ultrasound was inconclusive, and repeat X-ray revealed pneumoperitoneum. Laparotomy found purulent peritonitis from a subcapsular abscess in segment II, perforating both diaphragmatic and visceral hepatic surfaces. *Enterococcus faecalis* was isolated, and antibiotics administered. A secondary abscess in segment VII was later drained percutaneously. The patient recovered after 37 days. This case highlights the diagnostic challenge of ruptured PLA and the value of combined surgical and minimally invasive management.

## Introduction

Pyogenic liver abscess (PLA) is an uncommon but serious infection with mortality rates of 10%–19% [1]. Risk factors include diabetes, biliary disease, cirrhosis, and malignancy [1]. The most frequent pathogens are *Klebsiella pneumoniae* and *Escherichia coli*, while *Enterococcus faecalis* is rarely reported [1, 2]. Spontaneous rupture of PLA occurs in approximately 3.8%–5.4% of cases [3, 4]. Mortality is substantially higher than in non-ruptured PLA, with historical reports up to 43.5% [4], although outcomes have improved in recent series with prompt surgical intervention [3]. Ruptured PLA may present with pneumoperitoneum and peritonitis, mimicking perforated hollow viscus [1, 5].

Here, we report a rare case of ruptured PLA caused by *E. faecalis*, initially misdiagnosed as perforated gastric ulcer, emphasizing the diagnostic challenges and the importance of combining open and minimally invasive management.

## Case presentation

A 48-year-old male with known gastric ulcer disease and no other comorbidities was admitted to a surgical clinic with upper gastrointestinal bleeding. Endoscopy revealed a stable coagulum at the fundus–corpus junction and a 10–12 mm anterior wall ulcer with a retracted visible vessel (Forrest IIa–IIb), treated with injection hemostasis. The patient remained stable and was discharged on day 4.

Forty-eight hours later, he presented to the emergency department with fever up to 40°C, malaise, nausea, and abdominal fullness without vomiting or abdominal pain. He denied gastrointestinal bleeding. Laboratory tests revealed leukocytosis of 21 × 10^9^/L with 87% neutrophils, hemoglobin 106 g/L, bilirubin 24/17 μmol/L, AST 204 U/L, ALT 262 U/L, CRP >32 mg/dl, and D-dimer 6121 ng/ml.

On admission, no peritoneal irritation was evident. Abdominal ultrasound showed a normal right hepatic lobe, while assessment of the left lobe was limited by bowel gas. The initial abdominal X-ray was unremarkable.

Within 2 hr of admission, the patient’s condition deteriorated rapidly, developing septic shock and peritoneal signs. Repeat abdominal X-ray revealed free subdiaphragmatic air. Given the acute deterioration and new findings, urgent laparotomy was indicated for suspected gastric perforation.

**Figure 1:**
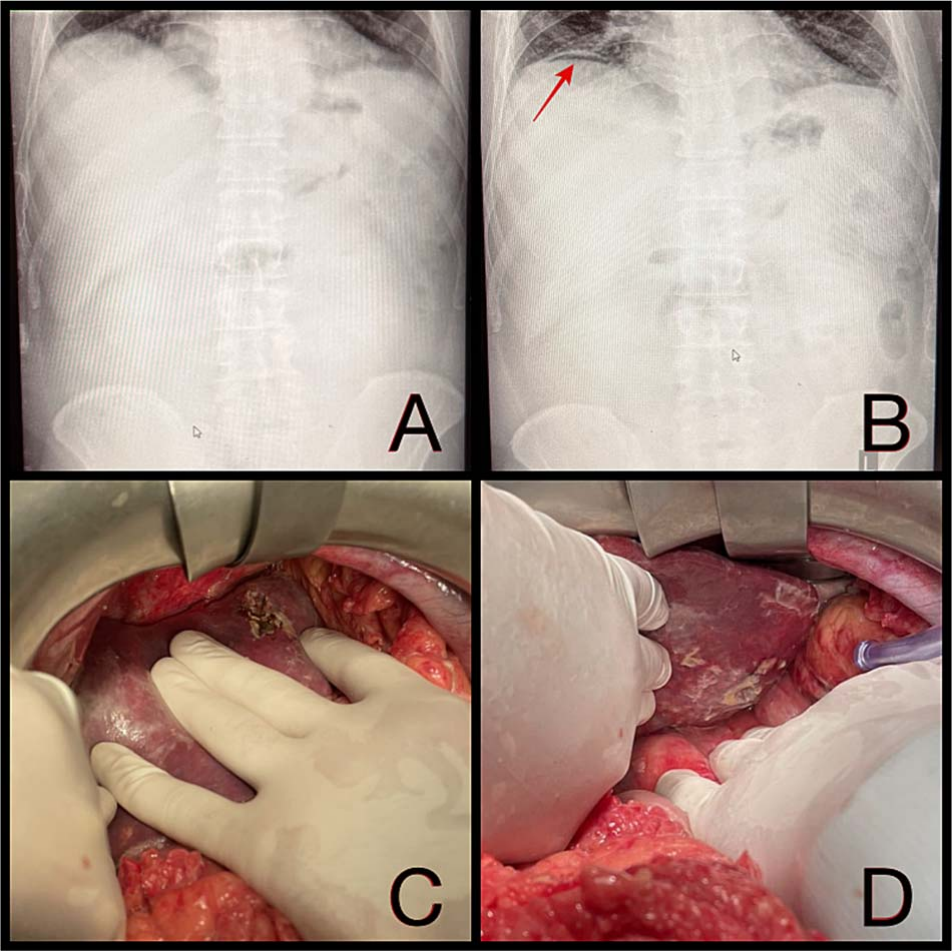
(A and B) Abdominal radiographs showing progression from no free gas (A) to a crescent of pneumoperitoneum beneath the right hemidiaphragm (B, arrow) within 2 hr. (C and D) Intraoperative findings of a ruptured pyogenic liver abscess in segment II, perforating both the diaphragmatic and visceral hepatic surfaces.

Intraoperatively, a ruptured liver abscess in segment II was found, perforating both the diaphragmatic and visceral hepatic surfaces. There was purulent discharge into the peritoneal cavity, predominantly in the upper abdomen. No gastrointestinal perforation was identified. Abdominal lavage, liver biopsy, and drainage were performed, followed by laparostomy (Fig. 1).

At second-look laparotomy after 48 hr, lavage demonstrated marked improvement. Five drains were placed and the abdomen was closed (laparosynthesis). Microbiological cultures isolated *E. faecalis*. Antibiotic therapy was tailored accordingly, starting with intravenous levofloxacin 500 mg twice daily.

**Figure 2:**
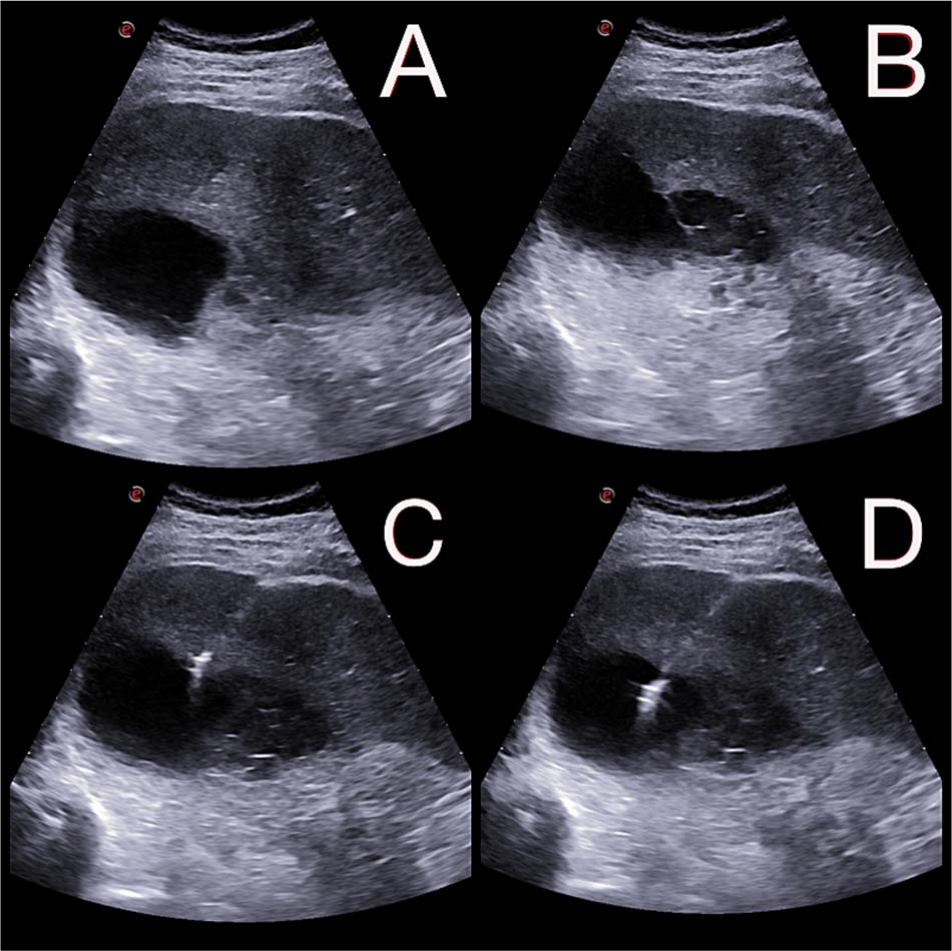
(A and B) Abdominal ultrasound depicting a 9 × 5 cm abscess in segment VII. (C and D) Percutaneous drainage performed with placement of a 10 Fr catheter under ultrasound guidance.

**Figure 3:**
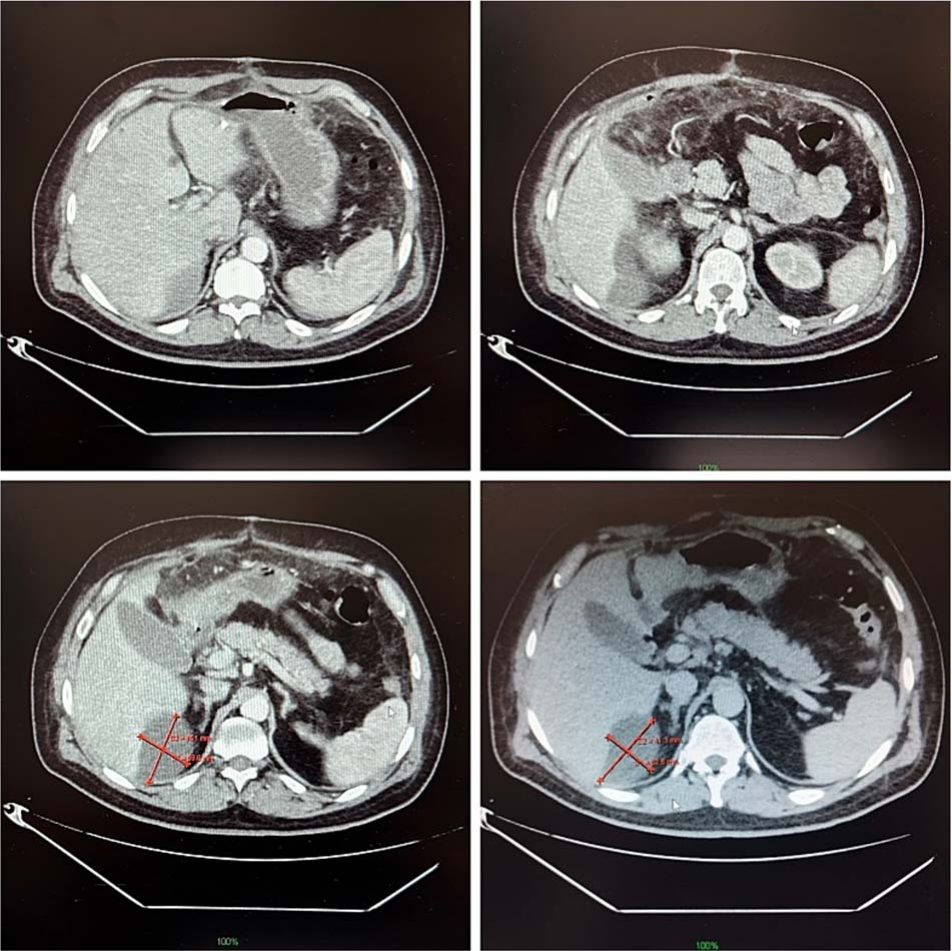
Contrast-enhanced abdominal CT on postoperative day 14, demonstrating a 9 × 5 cm abscess in segment VII.

On postoperative day 14, the patient remained febrile. Computed tomography revealed a secondary abscess in segment VII (Fig. 2), which was successfully managed by percutaneous drainage under ultrasound guidance (Fig. 3). The regimen was subsequently switched to intravenous linezolid 600 mg twice daily, combined with antifungal therapy. The drains were gradually removed by postoperative day 30, while intravenous therapy continued until day 35. The patient was discharged on day 37.

## Discussion

Rupture of PLA is infrequent but associated with worse outcomes. While historical series reported mortality up to 43.5%, recent cohorts describe improved survival rates (as low as ~4%) with prompt surgical management [3, 4].

Risk factors for rupture include large abscess size (>6 cm), cirrhosis, gas-forming organisms, and subcapsular or left-lobe location [3]. Wang *et al.* [6] identified diabetes and biliary disease as dominant risk factors for PLA.

In our patient, the abscess was located subcapsularly in the left lobe—a frequent risk factor for rupture—but exceptionally, it penetrated both diaphragmatic and visceral hepatic surfaces, a finding rarely documented in the literature. Enterococcal PLA is increasingly reported and has been associated with significantly higher treatment failure and mortality [7]; gas-forming PLA remain classically linked to *K. pneumoniae* [2]. Rarely, *Enterococcus* species have been implicated in gas-containing abscesses in other sites [8], making the etiology of our case unusual.

Possible mechanisms include bacterial translocation following gastric ulcer bleeding and endoscopic hemostasis, iatrogenic seeding via endoscopic equipment (as *Enterococcus* is known to persist in biofilms), or secondary biliary contamination. This underlines the importance of microbiological sampling and targeted therapy.

The diagnostic challenge was substantial. Pneumoperitoneum is most often caused by hollow viscus perforation [1]. In this case, repeat X-ray demonstrated free air, and urgent laparotomy was indicated. Similar misdiagnoses of ruptured PLA as gastric perforation have been described [5, 9]. Non-surgical treatment of ruptured PLA has been reported in selected stable patients without diffuse peritonitis [10, 11]. However, in cases presenting with septic shock and peritonitis, as in our patient, emergency laparotomy remains mandatory. Older age has been associated with prolonged hospitalization in PLA [12].

Spontaneous rupture can also extend toward atypical sites, such as the abdominal wall, forming secondary collections [13]. The subsequent development of a secondary abscess in segment VII in our case emphasizes the importance of close postoperative imaging. Multifocal disease or incomplete drainage are possible mechanisms, despite negative ultrasound findings in the right lobe and two-stage abdominal lavage. Management depends on the clinical scenario. In cases with diffuse peritonitis and septic shock, urgent laparotomy with lavage and drainage is required [3]. In stable patients with liver abscesses, including selected cases of localized rupture, percutaneous catheter drainage plus antibiotics can be effective [14]. In our patient, a hybrid approach—emergency laparotomy followed by percutaneous drainage of a secondary abscess—proved successful and reflects the modern trend of integrating open and minimally invasive therapies.

The patient recovered after 37 days. This case highlights the diagnostic challenge of ruptured PLA and the importance of combined surgical and minimally invasive management.

## References

[1] Sharma S, Ahuja V. Liver abscess: complications and treatment. Clin Liver Dis (Hoboken) 2021;18:122–6. 10.1002/cld.1128 34691398 PMC8518335

[2] Zhang S, Zhang X, Wu Q, et al. Clinical, microbiological, and molecular epidemiological characteristics of *Klebsiella pneumoniae*-induced pyogenic liver abscess. Antimicrob Resist Infect Control 2019;8:166. 31673355 10.1186/s13756-019-0615-2 PMC6819602

[3] Jun CH, Yoon JH, Wi JW, et al. Risk factors and clinical outcomes for spontaneous rupture of pyogenic liver abscess. J Dig Dis 2015;16:31–6. 10.1111/1751-2980.12209 25385432

[4] Chou FF, Sheen-Chen SM, Lee TY. Rupture of pyogenic liver abscess. Am J Gastroenterol 1995;90:767–70. 10.1111/j.1572-0241.1995.tb09316.x 7733086

[5] Pham Van T, Vu Ngoc S, Nguyen Hoang NA, et al. Ruptured liver abscess presenting as pneumoperitoneum caused by *Klebsiella pneumoniae*: a case report. BMC Surg 2020;20:228. 10.1186/s12893-020-00858-w 33028298 PMC7542763

[6] Wang JL, Hsu CR, Wu CY, et al. Diabetes and obesity and risk of pyogenic liver abscess. Sci Rep 2023;13:7922. 10.1038/s41598-023-34889-z 37193729 PMC10188555

[7] Oliosi E, Rossi G, Nguyen Y, et al. Enterococcal pyogenic liver abscesses: high risk of treatment failure and mortality. Eur J Clin Microbiol Infect Dis 2023;42:193–9. 10.1007/s10096-022-04543-z 36596905

[8] Park JH, Kim KH, Lee JH, et al. Gas-containing epidural abscess caused by *Enterococcus faecalis*. Neurospine 2015;12:139–42.

[9] Lai YC, Su YJ, Chang WH. Ruptured hepatic abscess mimicking perforated viscus. Int J Infect Dis 2008;12:e95–7. 10.1016/j.ijid.2008.06.005 18768341

[10] Motoyama T, Ogasawara S, Chiba T, et al. Successful non-surgical treatment of ruptured pyogenic liver abscess. Intern Med 2013;52:2619–22. 10.2169/internalmedicine.52.0980 24292751

[11] Lee KJ, Ryu SH. Ruptured gas-forming pyogenic liver abscess into the peritoneal cavity treated successfully with medical treatment. Korean J Gastroenterol 2018;71:45–8. 10.4166/kjg.2018.71.1.45 29361813 PMC12285798

[12] Chan KS, Junnarkar SP, Low JK, et al. Aging is associated with prolonged hospitalisation stay in pyogenic liver abscess. Malays J Med Sci 2022;29:59–73. 10.21315/mjms2022.29.5.7 36474543 PMC9680999

[13] Zizzo M, Zaghi C, Manenti A, et al. Abdominal wall abscess secondary to spontaneous rupture of pyogenic liver abscess. Int J Surg Case Rep 2016;25:110–3. 10.1016/j.ijscr.2016.06.026 27351622 PMC4925907

[14] Haider SJ, Tarulli M, McNulty NJ, et al. Liver abscesses: factors that influence outcome of percutaneous drainage. AJR Am J Roentgenol 2017;209:205–13. 28504550 10.2214/AJR.16.17713

